# A Cross‐Sectional Analysis of Traumatic Experiences, Post‐Traumatic Stress Disorder Symptoms and Chronic Pain in Northern Ireland

**DOI:** 10.1002/ejp.70044

**Published:** 2025-05-29

**Authors:** Kevin E. Vowles, Christina Mallett, Jason Brooks, Emma Berry, Danielle Rainey, Claire Briggs, Maura McCarron

**Affiliations:** ^1^ School of Psychology Queen's University Belfast Belfast UK; ^2^ Centre for Pain Rehabilitation Belfast Health and Social Care Trust Belfast UK; ^3^ Adult Psychological Therapies Service Western Health and Social Care Trust Derry UK

**Keywords:** chronic pain, co‐morbidity, Northern Ireland, post‐traumatic stress disorder (PTSD), traumatic experiences

## Abstract

**Background:**

Chronic pain and post‐traumatic stress disorder (PTSD) are frequently comorbid and are associated with more significant pain‐related disruption than chronic pain alone. It is not clear if these disruptions are due to traumatic experience or specific symptoms related to PTSD.

**Methods:**

This issue was evaluated in a large sample (*N =* 1367) of individuals with chronic pain presenting for treatment at an interdisciplinary pain rehabilitation service. As a secondary objective, the comorbidity of chronic pain and PTSD in Northern Ireland (NI) was also examined given high regional prevalence rates. Participants completed a PTSD screening measure, along with measures of pain interference, social functioning, pain anxiety, pain self‐efficacy, pain intensity and depression.

**Results:**

Screening indicated that 46.4% had never experienced a traumatic event, 22.5% had experienced a traumatic event but screened negative for PTSD and 31.1% screened positive for PTSD. Following identification of covariates, a Multivariate Analysis of Covariance examined differences in dependent measures by PTSD category, which yielded a similar pattern of results across measures. The group that screened positive for PTSD reported worse functioning and more disruptions in comparison to the other two PTSD groups, with the latter groups not differing on any measure.

**Conclusions:**

These analyses indicate that poorer functioning was not associated with trauma exposure alone; rather, it was experienced in association with PTSD symptoms. Further, comorbidity rates of PTSD and chronic pain in this NI dwelling sample were at the high end of the range in relation to previous work and exceeded past year regional prevalence estimates.

**Significance:**

PTSD assessment in those with chronic pain may be best served by evaluating the impact of these experiences on function, rather than focusing on the traumatic experiences in isolation. Further, there is scope to develop integrated chronic pain and PTSD treatments.

## 1 Introduction

Chronic pain and post‐traumatic stress disorder (PTSD) frequently co‐occur. Two separate literature reviews calculated a pooled comorbidity prevalence rate of approximately 10% (Fishbain et al. 2017; Siqveland et al. 2017), greater than the population's lifetime PTSD prevalence rate of 6.8% (Harvard Medical School 2017). Although PTSD prevalence varies across chronic pain studies (0%–57%), the presence of PTSD is reliably associated with increased emotional and physical disruption (Fishbain et al. 2017; Reed et al. 2024).

There are approaches to this issue which prioritise traumatic event experience as the key risk. For example, Felitti et al. (1998) found that recall of multiple traumatic experiences during childhood was related to a variety of healthcare risks during adulthood. The focus of these approaches has broadened out to include a lifetime history of traumatic events, collectively referred to as trauma informed care (TIC) within both systematic reviews (Reeves 2015) and government policy (Office for Health Improvement and Disparities 2022; Substance Abuse and Mental Health Services Administration 2014). The TIC model of aetiology emphasises exposure to traumatic stressors as a primary health risk (DeCandia and Guarino 2015).

In contrast, a diagnosis of PTSD requires both a precipitating traumatic event and clinically significant disturbances in mood and behaviour (American Psychiatric Association 2013). Specifically, four symptom clusters are required, including intrusive symptoms, avoidance of trauma‐associated stimuli, negative mood and increased trauma‐related arousal. There is also a required symptom duration of at least 1 month.

There is likely a meaningful difference between the experience of trauma and the experience of PTSD symptoms in those with chronic pain. To our knowledge, this issue has only been examined in one study by Åkerblom et al. (2017) who found that the presence of PTSD was associated with greater emotional and functional difficulties than in those with trauma exposure alone. That pattern of findings suggests it is more clinically useful to assess PTSD than trauma experiences alone.

This issue was examined in the present study by evaluating pain‐related functioning in a large sample of treatment‐seeking individuals with chronic pain: (1) without a history of traumatic experience, (2) with previous traumatic experience and without significant PTSD symptoms and (3) with both previous experience and significant PTSD symptoms. Such an evaluation would clarify whether trauma exposure, in and of itself, was related to greater distress and disability or if the experiences symptomatic of PTSD diagnosis were a key consideration.

In addition, these issues were examined in Northern Ireland (NI), a region which experienced significant civil conflict during the latter half of the 20th century. Northern Ireland has some of the highest rates of PTSD in Europe (Ferry et al. 2014) with Bunting et al. (2013) reporting that lifetime prevalence was 61% for traumatic experience and 9% for PTSD. Furthermore, chronic pain is common as it was the most prevalent long‐term condition identified in the 2021 government census (Northern Ireland Statistics and Research Agency 2022). Thus, an examination of trauma exposure and PTSD symptoms in this region represented an opportunity with high potential for community relevance.

## 2 Method

### 2.1 Participants

Data were collected between December 2021 and October 2024 from consecutive adults with chronic pain presenting for treatment to the Belfast Centre for Pain Rehabilitation, a secondary care interdisciplinary treatment service based within the Belfast Health and Social Care Trust of the United Kingdom's (UK) National Health Service (NHS). Upon referral to the service, all individuals were sent a secure online link to complete the clinic's self‐report assessment battery, which evaluated demographic and pain background details, treatment history, psychosocial functioning and pain‐related impacts. For those who were unable to complete the survey online, paper copies were provided and the Centre's nursing team provided assistance where required, for example, in the case of reading or vision difficulties. Patient responses were scored and evaluated by a member of the clinic staff to aid in initial treatment decisions.

Approval was also in place to use these clinical data in research (NHS Research Ethics Committee reference number: 313982). Participants were provided with an information sheet in relation to research activities and were asked to provide informed consent that their de‐identified data could be used for research purposes. Over the study period, 1541 individuals entered the service and 92.7% (*n =* 1428) provided informed consent.

Of those who provided consent, 60.0% were female, 34.3% were male and 5.7% declined to provide details. Participant age averaged 51.9 years (SD = 14.5) and years of education averaged 13.1 years (SD = 3.5). Regarding ethnicity, 83.6% were White, 1.6% were of mixed ethnicity, 0.9% were Black, 0.7% were Asian and the remaining 13.2% declined to provide details. In terms of relationship status, 33.3% were married or in a registered civil partnership, 29.8% were single, 6.9% were separated, 9.4% were divorced, 5.1% were widowed and 15.5% declined to provide details.

Average pain duration was 12.3 years (SD = 10.1). The most frequent pain locations were lower extremity, 89.0%, and low back, 88.1%, followed by neck/upper back, 67.2%, upper extremity, 66.6%, abdomen/pelvic, 43.1% and head, 39.1%. The average number of pain locations noted was 4.1 (SD = 1.5) with only 6.3% of participants noting a single location. In terms of previous pain treatments, 80.6% had physiotherapy, 50.4% had injections and 27.9% had surgery. For each of these previous pain treatments, a minority noted that they had found them helpful, 10.9%, 16.8% and 26.5%, respectively.

### 2.2 Measures

The questionnaire battery included several standardised measures related to pain and pain‐related functioning, as well as questions in relation to demographic and pain background and history. Specific measures are detailed below. In these data, internal consistency across all measures was excellent, Cronbach's *α* range: 0.83–0.98.

#### 2.2.1 Trauma Experience and Trauma Symptoms

The Primary Care PTSD Screen for the Diagnostic and Statistical Manual of Mental Disorders (PC‐PTSD; Prins et al. 2016) was used to assess lifetime traumatic experience and PTSD symptoms. The measure's items reflect the Diagnostic and Statistical Manual of Mental Disorders—5 criteria for PTSD (American Psychiatric Association 2013).

There are six items in total, including a single dichotomous (yes/no) item that asks respondents about lifetime traumatic event exposure (e.g., a serious accident/fire, physical or sexual abuse, a natural disaster, war, witnessing someone being killed or seriously injured or losing a loved one to suicide or homicide). If a respondent indicates no trauma exposure, the questionnaire is concluded. When respondents report trauma exposure, five further dichotomous (yes/no) items assess for symptoms related to a diagnosis of PTSD that have occurred over the preceding month, including nightmares, avoidance, hypervigilance, feeling numb/detached and guilt/blame of self or others. Responses are summed to calculate a total ranging from 0 to 5.

Evaluations of the PC‐PTSD have indicated that a score of 4 or more is an optimal point with regard to maximising PTSD diagnostic accuracy. Sensitivity and specificity for this cut point were 0.78 and 0.91, respectively, in a US veterans sample in relation to structured clinical interview (Bovin et al. 2021) and 1.0 and 0.85, respectively, in a non‐military primary care sample in relation to consensus diagnosis following clinical interview with a family medicine medical doctor and clinical psychologist (Williamson et al. 2022).

In the present analysis, individuals were therefore grouped into one of three PTSD categories based on their response to the PC‐PTSD. Those who indicated no lifetime trauma exposure were categorised as *No Event*, those with trauma exposure and symptom scores of three or less were categorised as *Event, no PTSD*, and those with trauma exposure and symptom scores of four or more were categorised into the *Probable PTSD* group.

#### 2.2.2 Pain Interference

The Patient Reported Outcomes Measurement Information System (PROMIS), Pain Interference—Short Form 8a was used to assess the impact of pain on daily functioning (Amtmann et al. 2010). The measure included eight items, which ranged from 1 (not at all) to 5 (very much), which were summed and then converted to *t*‐scores (mean: 50; SD: 10) with higher scores indicating greater pain interference. The PROMIS Pain Interference measures have broad psychometric support across a range of chronic pain samples (Amtmann et al. 2010; Askew et al. 2016).

#### 2.2.3 Social Role Functioning

The PROMIS, Ability to Participate in Social Roles and Activities—Short Form 8a was used to evaluate ability to engage in usual social roles and activities (Hahn et al. 2010). As with the PROMIS Pain Interference measure, responses on the eight items ranged from 1 (not at all) to 5 (very much), which were summed and then converted to t‐scores (mean: 50; SD: 10). For this measure, higher scores were indicative of better social functioning. The Social Role PROMIS measure has demonstrated excellent psychometric properties (Terwee et al. 2018).

#### 2.2.4 Pain‐Related Anxiety

The four‐item version of the Pain Anxiety Symptoms Scale (PASS‐4; Vowles et al. 2024) was used to assess pain‐related anxiety. The PASS‐4 was derived from the widely used longer versions of the PASS (McCracken et al. 1992; McCracken and Dhingra 2002) using Item Response Theory analyses to identify the four best‐performing items across a large multisite sample of individuals with chronic pain (Vowles et al. 2024). The PASS‐4 items are rated on a 6‐point scale from never (0) to always (5), with summed scores ranging from 0 to 20 and higher scores indicating higher pain‐related anxiety. The initial validation study indicated strong psychometric properties in terms of internal consistency and reliable relations with other measures of pain‐related functioning.

#### 2.2.5 Pain Self‐Efficacy

The two‐item Pain Self‐Efficacy Questionnaire (PSEQ‐2, Nicholas et al. 2015) was used to evaluate participant confidence in carrying out activities while experiencing pain. Items are rated on a six‐point scale from 0 (not at all confident) to 6 (completely confident). The items are summed to yield a total score that ranges from 0 to 12, with higher scores indicating greater confidence. Scores on the PSEQ‐2 are reliably associated with aspects of pain and pain‐related functioning (Dubé et al. 2021; Nicholas et al. 2015).

#### 2.2.6 Usual Pain Intensity

A 0 (no pain) to 10 (pain as severe as it could be) Numerical Rating Scale was used to assess average pain over the past week. This method of assessing average pain is widely used and established (Campbell and Vowles 2008; Nicholas et al. 2019).

#### 2.2.7 Depression

Two measures of depression were used in these analyses, as the service used the Patient Health Questionnaire‐9 (PHQ‐9; Kroenke et al. 2001) from database inception through October 2023. Following that date, the PROMIS Depression—Short Form 4a was used (Pilkonis et al. 2011).

The PHQ‐9 was used by 911 participants. On this measure, symptom frequency of depression is rated on items that range from 0 (not at all) to 3 (nearly every day). Items are summed with scores ranging from 0 to 24, and higher scores indicating greater frequency. The PHQ‐9 has been widely used in a variety of healthcare settings and has established reliability and validity (Negeri et al. 2021).

For the remaining 456 participants, the PROMIS Depression was used, which included four items that assess depression symptom frequency ranging from 1 (never) to 5 (always). Item scores were summed and converted to t‐scores with higher scores indicating a more frequent experience of symptoms. The PROMIS Depression measures have been widely used since their development and have strong evidence in terms of psychometric properties and clinical utility (Kroenke et al. 2021; Schalet et al. 2016).

### 2.3 Analytic Approach

Four analytic steps were planned. First, data integrity evaluations were performed, including evaluation of data distribution, outliers, patterns of missing data and internal consistency of dependent measures.

Second, participants were categorised in relation to PTSD status. Because there are established gender differences in rates of chronic pain, pain‐related functioning, experience of traumatic events and development of PTSD (Bartley and Fillingim 2013; Harvard Medical School 2017), an initial chi‐square analysis was performed to determine if there were different gender distributions within the PTSD category. If differences were indicated, we planned to conduct additional analyses separately for each gender.

Third, two multivariate analysis of variance (MANOVA) were performed to identify relevant covariates to include in our primary analyses examining differences in functioning based on PTSD status. The first analysis examined gender differences in dependent measures, including pain interference, social role functioning, pain‐related fear, pain self‐efficacy and pain intensity. Next, we examined differences in demographic and pain‐related background variables based on PTSD category, including age, number of years of education, number of pain locations (1–6) and pain duration. We planned to follow up significant MANOVA results with univariate analysis of variance (ANOVA), and, if warranted, to follow significant ANOVAs up with Bonferroni‐corrected pairwise tests.

Fourth, the primary analyses examined differences in functioning based on PTSD category. A MANCOVA examined differences in five of the six dependent variables, including pain interference, social role functioning, pain anxiety, pain self‐efficacy and usual pain intensity (past week) based on PTSD status. For our final dependent variable, depression, two separate ANCOVAs were performed, one for the PHQ‐9 and another for the PROMIS Depression. We planned that all primary analyses would include the covariates identified in the previously detailed analytic steps.

Data management and descriptive analyses used the Statistical Package for Social Sciences, version 29 (IBM Corp 2022). All other analyses were done using R Studio (Build 524, RStudio Team 2023).

## 3 Results

### 3.1 Data Integrity Evaluation

Several separate data integrity evaluations were performed. Initial inspections of distributions indicated that the data for 61 individuals were univariate outliers on one or more dependent measures, including pain interference (*n =* 13), social role functioning (*n* = 26), pain‐related anxiety (*n* = 35) and pain intensity (*n* = 12). Visual inspection of both participant data and boxplots indicated that these outlying scores were all at or close to the lowest range of severity for each measure. Individuals with outlying data were removed from further consideration, thus analyses proceeded with data from 1367 individuals (95.7% of the initial dataset). Missing data were rare, as the clinical data collection system emphasises the need for complete information and flags missing responses to request patient completion. In total, eight individuals failed to provide pain intensity information, and all other data were fully complete. Patterns of data missingness were therefore not evaluated, and a maximum likelihood approach was used to include all available data.

Following removal of univariate outliers, no multivariate outliers were identified upon evaluation of Mahalanobis Distance. Data were non‐kurtotic, with an absolute value of < 0.73 for all kurtosis statistics, although Shapiro–Wilk tests indicated non‐normality among all the dependent variables (all *p* < 0.05). Further, a Box's *M* test to evaluate homogeneity of covariance matrices for the dependent measures was significant, Box's *M* = 103.1, *p <* 0.001. Therefore, Pillai's Trace was used as the multivariate test statistic in all MANOVA analyses followed by Welch's one‐way ANOVAs given that they yield robust results when there is evidence of non‐normality and heterogeneity in covariance, as well as when cell sizes differ, as we expected them to in relation to PTSD status (Ateş et al. 2019; Delacre et al. 2019).

### 3.2 Preliminary Analyses

When individuals were classified by PTSD status, approximately half of the sample, 46.4% (*n* = 662), reported no previous experience of a traumatic event (*No Event*). In the remaining sample, 22.5% (*n* = 322) experienced a traumatic event but screened negative for PTSD (*Event*, *no PTSD*), and 31.1% (*n* = 444) experienced a traumatic event and screened positive for PTSD (*Probable PTSD*).

There were few significant gender differences. Two chi‐square analyses were performed, one including three gender categories (female, male and prefer not to say) and the other two gender categories (female and male). Results for both analyses were not significant, χ^2^ (4, *n =* 1367) = 7.4, *p* = 0.12 and χ^2^ (2, *n =* 1292) = 5.3, *p* = 0.07, respectively. Gender proportions within each PTSD category are shown in Table 1, which also shows the female: male proportion for each category. As shown in that table, for every male, there were 2.1 females in the *No Event* category, 1.6 females in the *Event, no PTSD* category and 1.5 females in the *Probable PTSD* category. Given the lack of gender differences in terms of PTSD category, a single analysis was performed that included both genders.

**TABLE 1:** Gender by PTSD status grouping information.

Gender	No event	Event, no PTSD	Probable PTSD
Female	392 (63.2%)	187 (59.9%)	247 (56.8%)
Male	191 (30.8%)	113 (36.3%)	162 (37.2%)
Prefer not to say	37 (6.0%)	12 (3.8%)	26 (6.0%)
Proportion female:male	2.1:1	1.6:1	1.5:1

*Note:* *N* = 1367.

The MANOVA examining differences between males and females in dependent measures was significant, Pillai's = 0.01, *F* (5, 1278) = 2.4 *p* ≤ 0.05, although follow‐up Welch's ANOVAs indicated significant gender differences for pain self‐efficacy only, *F* (1, 1282) = 4.7, *p* < 0.05. Descriptively, female average pain self‐efficacy was higher than male, 2.7 (SD: 2.9) and 2.0 (SD: 2.9), respectively. All other ANOVAs, including pain interference, social role functioning, pain‐related anxiety and pain intensity, were not significant, all *F* (1, 1282) < 1.9, all *p* > 0.17.

The MANOVA evaluating demographic and pain background differences based on PTSD category was also significant, Pillai's = 0.06, *F* (8, 2678) = 11.2, *p* ≤ 0.001. Follow‐up Welch's ANOVAs were significant for age and total number of pain locations, both *F* (2, 1341) > 17.8, both *p* < 0.001. With regard to age, the *No Event* group was significantly older than the other groups, with an average age of 54.4 years (SD: 16.0). The other two groups did not significantly differ in age, 50.3 years (SD: 14.1) for *Event, no PTSD* and 49.3 years (SD: 11.5) for *Probable PTSD*. Regarding the number of pain locations, those with *Probable PTSD* reported more pain sites on average, 4.5 (SD: 1.5) than the other two groups. The other two groups did not differ significantly in number of pain sites, 3.8 (SD: 1.5) for *No Event* and 4.1 (SD: 1.5) for *Event, no PTSD*. There were no significant differences indicated for years of education or for pain duration by PTSD category, both *F* (2, 1341) < 1.6, both *p* > 0.20.

### 3.3 Evaluation of Differences in Functioning by PTSD Category

Given the preliminary analyses, the primary MANCOVA consisted of a single analysis, which included three covariates (gender, age and number of pain locations). Only two genders were included (males and females) as a gender of ‘prefer not to say’ was not clear enough for analysis and a dichotomous gender variable would yield interpretable MANCOVA results.

The MANOVA of dependent measures by PTSD category indicated a significant omnibus effect, Pillai's = 0.11, *F* (1, 2532) = 15.2, *p* ≤ 0.001, as well as significant effects for all three covariates, gender, Pillai's = 0.01, *F* (5, 1265) = 2.3, *p* ≤ 0.05; age, Pillai's = 0.03, *F* (5, 1265) = 7.5, *p* ≤ 0.001, and number of pain locations, Pillai's = 0.06, *F* (5, 1265) = 14.7, *p* ≤ 0.001. Follow‐up one‐way Welch's ANCOVAs indicated a significant effect of PTSD category for all dependent measures, all *F* > 8.0, all *p* < 0.001. A significant covariate effect of gender was only present for pain self‐efficacy, *F* (1, 1269) = 4.2, *p* < 0.05, and non‐significant for all other dependent measures, all *F* < 1.0, all *p* > 0.33. Significant covariate effects for age and pain location were indicated across almost all dependent measures, all *F* > 8.4, all *p* < 0.005. The sole exception was for pain anxiety, for which there was no covariate effect of age, *F* (1, 1269) = 0.001, *p* = 0.98.

Finally, pairwise analyses were performed with a Bonferroni correction for multiple comparisons. Across all comparisons, an identical pattern of findings was indicated. In each case, the *Probable PTSD* group significantly differed from the other two groups, *No Event* and *Event, no PTSD*, while these latter two groups did not differ significantly from one another. In each case, the *Probable PTSD* group noted greater symptom impact on functioning and greater symptom severity. Estimated marginal means (EMMs) are in Table 2. Note that EMM calculation excluded all non‐significant covariates, as indicated in the above detailed ANCOVA results.

**TABLE 2:** Estimated marginal means (SE) for Bonferroni‐controlled pairwise comparisons.

	PTSD category		
	No event	Event, no PTSD	Probable PTSD
Pain interference	70.4 (0.2)^a^	71.0 (0.3)^a^	72.8 (0.3)^b^
Social role functioning	33.5 (0.2)^a^	33.2 (0.3)^a^	30.5 (0.3)^b^
Pain‐related fear	15.4 (0.1)^a^	15.3 (0.2)^a^	17.2 (0.2)^b^
Usual pain intensity (past wk.)	8.1 (0.1)^a^	8.0 (0.1)^a^	8.3 (0.1)^b^
Pain self‐efficacy	3.3 (0.1)^a^	3.5 (0.2)^a^	1.9 (0.1)^b^
Depression			
PHQ‐9	16.0 (0.3)^a^	16.9 (0.4)^a^	21.6 (0.4)^b^
PROMIS (4a)	63.1 (0.8)^a^	64.9 (0.9)^a^	72.2 (0.9)^b^

*Note:* Different superscripts indicate significant pairwise differences.

For the Welch's ANCOVAs of the two depression measures, the pattern of findings was the same as for all other measures—the *Probable PTSD* group had higher scores than the other two groups, who did not differ from one another, both *F* > 38.3, both *p* < 0.001. Table 2 also has EMMs for both measures of depression. For the PHQ‐9, age and number of pain locations were significant covariates, and for the PROMIS depression measure, only number of pain locations was a significant covariate. Table 3 displays specific pairwise comparisons and 95% confidence intervals between the *Probable PTSD* group and each of the other two groups. The overall pattern of findings in this Table illustrates the magnitude of group differences, ranging from fairly minor differences in pain intensity to large differences for both measures of depression.

**TABLE 3:** Descriptive details comparing Probable PTSD to other groups.

Variable	Comparison to probable PTSD	
	(Estimated marginal mean difference (SE); 95% confidence interval^a^)	
	No event	Event, no PTSD
Pain interference	−2.4 (0.3); −1.6 to −3.2	−1.8 (0.4); −0.8 to −2.7
Social role functioning	2.9 (0.4); 2.0 to 3.9	2.6 (0.4); 1.6 to 3.7
Pain‐related fear	−1.8 (0.2); −1.2 to −2.4	−2.0 (0.3); −1.3 to −2.6
Usual pain intensity (past wk.)	−0.2 (0.1); −0.02 to −0.5	−0.3 (0.1); −0.05 to −0.6
Pain self‐efficacy	1.4 (0.2); 0.9 to 1.8	1.6 (0.2); 1.1 to 2.1
Depression		
PHQ‐9	−5.1 (0.5); −4.0 to −6.2	−4.6 (0.6); −3.3 to −5.9
PROMIS (4a)	−8.5 (1.2); −5.7 to −11.4	−7.4 (1.3); −4.3 to −10.5

^a^ Values include Bonferroni adjustment for multiple comparison.

**FIGURE 1:**
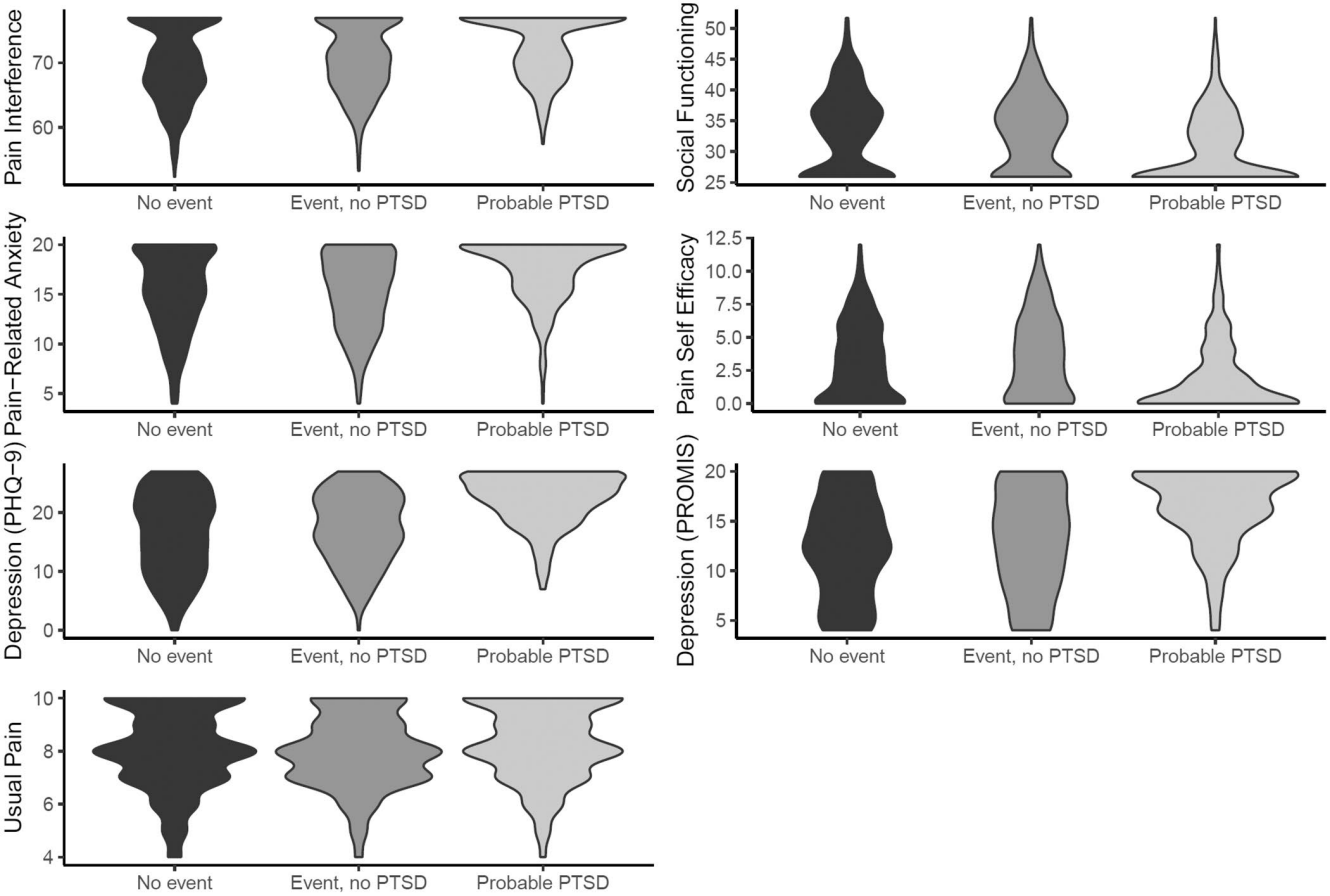
Violin plots for all dependent measures by PTSD category.

Figure 1 displays violin plots of mean scores for all groups on all dependent measures. As indicated in the figure, the *Probable PTSD* group's score distribution included fewer individuals in the low range and more individuals at the highest ranges.

## 4 Discussion

The above results are consistent with previous work in indicating that PTSD is present in a significant number of individuals with chronic pain and that this comorbidity is associated with greater emotional and functional disruption than chronic pain alone. In this study, the rate of *Probable PTSD* was 31.1%, which is within the range of rates reported in previous studies, albeit towards the higher end of prevalence, and substantially higher than the pooled rates of about 10% calculated within recent reviews (Fishbain et al. 2017; Siqveland et al. 2017). Thus, in this large sample of individuals presenting for chronic pain treatment, it is clear that PTSD is a clinically significant concern for a large proportion of individuals and, when present, is reliably associated with greater disruptions in emotional and physical functioning. Specifically, Probable PTSD seems a risk factor for added complexity across broad areas of living in a clinical population—people with chronic pain—who already present with significant complexity. Further, rates of PTSD in this sample are higher than in NI generally, where 12 months and lifetime prevalence rates were previously reported as 5.1% and 8.8%, respectively (Bunting et al. 2013), suggesting this comorbidity is of particular concern in the province.

In relation to our primary study aim, these data clearly indicate that the presence of lifetime traumatic experiences alone, at least as assessed here, was not related to more severe symptoms. Specifically, there were no statistically significant differences present between the *No Event* and *Event*, *no PTSD* groups on any of our dependent measures, including pain interference, social role functioning, pain‐related anxiety, self‐efficacy, pain intensity or depression. As far as we are aware, only one previous Swedish study has examined this issue, and it also found this pattern of results (Åkerblom et al. 2017). It will be worth performing similar analyses in future work to see if this pattern of findings is replicable in additional groups of individuals. If it is a reliable finding, that suggests clinical efforts may be best directed towards the assessment of PTSD symptoms specifically and less directed towards the assessment of traumatic experiences alone. That assertion is not intended to argue that the experience of trauma isn't important, but it may be that it isn't strongly predictive of ongoing emotional and functional difficulties, as there are many ways that individuals respond to trauma which can minimise or eliminate long‐term impact on quality of life and health (Hayes et al. 1996; Park 2010; Santiago et al. 2013).

The overall pattern of scores, as shown in the violin plots (Figure 1), provides an interesting insight into score distribution between the three groups. For the most part, the distributions for the *No Event* and *Event*, *no PTSD* groups are similar enough in shape to be largely indistinguishable from one another based on visual inspection. In contrast, the *Probable PTSD* group has a pattern that is clearly distinct, with distributions largely weighted towards the severe range across all measures. In fact, the distributions indicate a potential floor or ceiling effect in scores. While the MANCOVA results indicate that there are between‐group differences, the distributions shown in the figure add further detail in terms of the pattern of difference and overall severity in the *Probable PTSD* group. The only exception to this pattern was for pain intensity, where the distributions are visually similar. While the same pattern of between‐group differences was indicated in ANCOVA, the magnitude of difference in pain intensity appears smaller between groups. These smaller differences in pain intensity between those with chronic pain with or without PTSD have been found in other studies as well (Giummarra et al. 2017; Lehinger et al. 2021), suggesting pain intensity may not be as salient a consideration in comparison to the other domains assessed here.

There are treatment considerations as well in relation to comorbid PTSD and chronic pain, as intervention traditionally tends to occur either sequentially or in parallel, presumably within different treatment services and with different providers (Lumley et al. 2022; Otis et al. 2009). Clinical experience suggests that treatment gains may be limited in both circumstances, as individuals will continue to experience difficulties for the untreated condition that negatively impact beneficial response to the treated one, may receive inconsistent messages or interventions across two treatments that end up diminishing benefit from both, or may never begin treatment based on the assumption that the other condition must be successfully treated first. Thus, the chances of clinical utility are likely to be improved with interprofessional working, shared clinical models of care, and, perhaps most importantly, treatments that are integrated in terms of conceptualisation, approach and personnel.

There are few studies of integrated treatments, with only a pilot study of unidisciplinary psychological treatment in US military veterans showing evidence of feasibility and potential for benefit (Otis et al. 2009). There has been examination of PTSD symptoms following interdisciplinary chronic pain rehabilitation programmes, which are integrated and comprehensive interventions that could conceivably reduce PTSD impacts on functioning via the same change mechanisms to reduce pain‐related impacts on functioning, even though PTSD symptoms were not targeted specifically for change. Results of these programmes on PTSD symptoms have been mixed; however, with one study finding evidence of a positive effect (Gilliam et al. 2020) and the other finding no such benefit (Åkerblom et al. 2020). Finally, a recently published trial for co‐morbid chronic headache and PTSD in US veterans found that unidisciplinary psychology treatment for chronic headaches was related to significant improvements for both pain interference and PTSD, while treatment for PTSD was associated with improvement in PTSD symptoms, but not for pain interference (McGeary et al. 2022). Further work here is needed to illuminate whether appropriate interdisciplinary pain rehabilitation is sufficient to alleviate both pain and PTSD‐related interference, or whether an integrated intervention, which addresses both issues, yields superior outcomes. Given the high rates of comorbidity, it would be beneficial to determine optimal routes of treatment when PTSD is present in those with chronic pain.

There are limitations to consider. First, we used a screening measure for PTSD and, while that measure has very strong evidence of sensitivity and specificity for a diagnosis of PTSD, the fact remains that it is a brief assessment including only dichotomous items. Thus, the prevalence rate documented here is best viewed as an estimate until more thorough assessment details are collected and evaluated. That issue may be particularly relevant to the assessment of traumatic experiences, as the dichotomous item of the measure used did not allow for assessment regarding important characteristics of these experiences such as frequency, duration and intensity, nor an evaluation of cumulative burden. Options for more thorough PTSD assessment include questionnaires such as the PTSD Checklist (Blevins et al. 2015) or structured clinical interview (e.g., Weathers et al. 2018), with the latter being recognised as the diagnostic gold standard (Spoont et al. 2013). Second, these were cross‐sectional analyses using self‐report measures only, which limit any conclusions regarding directional relations and add potential for response bias. Finally, the collection of data in NI, while topical given the region's history and ongoing health concerns, means that these findings may not be generalisable to other regions with different characteristics.

To our knowledge, this is the largest study to date examining relations between PTSD and chronic pain, and the second to have assessed traumatic experience and experience of PTSD symptoms separately. It was also the first large‐scale analysis of chronic pain, pain‐related functioning and PTSD in NI. Overall, findings highlighted that almost one‐third of adults seeking treatment for chronic pain screened positive for *Probable PTSD*. Moreover, the study's findings strengthen the assertion that the experience of *Probable PTSD* can negatively influence the relation between pain‐related functioning and chronic pain. It also suggests that trauma exposure itself may not, in isolation, be a significant risk factor for poor functioning. Moreover, given the high rates of PTSD symptoms among individuals with chronic pain, globally and locally within our sample, integrative models of care appear necessary.

## Author Contributions

This study was designed by K.E.V., C.M., D.R., J.B. and E.B. K.E.V. and C.M. performed analyses with all authors critically evaluating the results. K.E.V. and C.M. co‐wrote the first draft of the manuscript, which was edited by D.R., J.B., E.B., C.B. and M.M. All authors have approved the final version of the manuscript.

## Conflicts of Interest

The authors declare no conflicts of interest.
